# Role of CD47 in Hematological Malignancies

**DOI:** 10.1186/s13045-020-00930-1

**Published:** 2020-07-16

**Authors:** Entsar Eladl, Rosemarie Tremblay-LeMay, Nasrin Rastgoo, Rumina Musani, Wenming Chen, Aijun Liu, Hong Chang

**Affiliations:** 1 Laboratory Medicine Program, Toronto General Hospital, University Health Network, University of Toronto, 11th floor, 200 Elizabeth Street, Toronto, ON M5G 2C4 Canada; 2 Department of Hematology, Beijing Chaoyang Hospital, Capital University, Beijing, China

**Keywords:** CD47, Immunotherapy, Targeted therapy, Hematological cancers

## Abstract

CD47, or integrin-associated protein, is a cell surface ligand expressed in low levels by nearly all cells of the body. It plays an integral role in various immune responses as well as autoimmunity, by sending a potent “don’t eat me” signal to prevent phagocytosis. A growing body of evidence demonstrates that CD47 is overexpressed in various hematological malignancies and its interaction with SIRPα on the phagocytic cells prevents phagocytosis of cancer cells. Additionally, it is expressed by different cell types in the tumor microenvironment and is required for establishing tumor metastasis. Overexpression of CD47 is thus often associated with poor clinical outcomes. CD47 has emerged as a potential therapeutic target and is being investigated in various preclinical studies as well as clinical trials to prove its safety and efficacy in treating hematological neoplasms. This review focuses on different therapeutic mechanisms to target CD47, either alone or in combination with other cell surface markers, and its pivotal role in impairing tumor growth and metastatic spread of various types of hematological malignancies.

## Background

### Structure and expression of CD47

CD47 is a heavily glycosylated 50 Kd cell surface protein belonging to the immunoglobulin family, originally named integrin-associated protein (IAP) [1]. It has an extracellular *N*-terminal IgV domain, five transmembrane domains, and a short C-terminal cytoplasmic tail. The three domains are variable between humans and animals in terms of total amino acid composition, giving four alternative isoforms [2]. CD47 is expressed by virtually all cells in the body, including those that do not express integrins, such as erythrocytes. Therefore, it is now more appropriate to refer to it as CD47 rather than IAP [3]. CD47 can be found in a bigger, more complex form associated with heparin and chondroitin sulfate glycosaminoglycan. This form is expressed in both human and murine T-cells as well as endothelial cells and is responsible for signal inhibition after binding of T-cells to thrombospondins (TSPs) [4].

### Mechanism of function and intracellular signalling

**Fig. 1:**
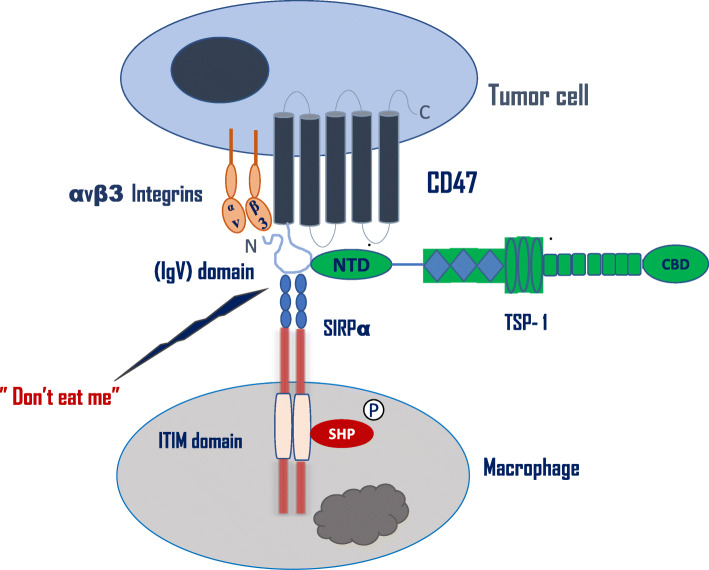
CD47 Structure and binding partners. CD47 is a transmembrane protein with 5 transmembrane domains, short intracytoplasmic C-terminal and *N*-terminal extracellular immunoglobulin variable (IgV) domain. Schematic diagram showing that CD47 can interact with αvβ3 integrin on the same cell or with SIRPα on the phagocytic cell through its IgV domain and activates “don’t eat me” signal. CD47 can also bind thrombospondin-1 (TSP) in the *N*-terminal, which promotes an interaction between CD47 and αvβ3 integrin and triggers αvβ3 integrin signaling

CD47 plays an essential role in various cellular functions including proliferation, apoptosis, adhesion, migration, and numerous immune responses. This occurs via cell-cell as well as cell-extracellular matrix interactions. Sick and colleagues published an exhaustive review of the biological functions of CD47 [5]. These functions are mediated through the binding of CD47 to its extracellular ligands such as signal regulatory proteins (SRPs) [6, 7] and thrombospondins (TSPs) [4, 5, 8]; membrane ligands such as integrins [9], vascular endothelial growth factor receptor-2 (VEGFR-2) [4], CD36 [8], and Fas (CD95) [10]; as well as intracellular ligands such as G_i_ proteins [11], BNIP3 [6], and Src and mitogen-activated protein kinases (MEK) [9] and protein 4.2 [12]. Figure 1 summarizes the CD47 structure and its ligands.

#### Interaction with extracellular ligands

##### SIRPα

is a transmembrane glycoprotein expressed on myeloid cells such as granulocytes, monocytes, macrophages, dendritic cells, and their precursors, including hematopoietic stem cells, as well as neuronal cells [13]. It is also known as CD172a, Src homology 2 domain-containing protein tyrosine phosphatase substrate-1 (SHPS-1) or brain immunoglobulin-like molecule with tyrosine-based activation motifs (BIT). It consists of an extracellular *N*-terminal domain composed of three immunoglobulin-like and a cytoplasmic domain, which has two tyrosine phosphorylation sites and four immunoreceptor tyrosine inhibitory motifs (ITIMs) [14].

Binding of CD47 to *N*-terminal of SIRPα on the phagocytic cells induces a phosphorylation reaction of the ITIM. This activates protein tyrosine phosphatases (PTPases) Src homology region 2 (SH2) domain-containing phosphatase1(SHP-1) and 2(SHP-2) [15]. Subsequently, dephosphorylation of immunoreceptor tyrosine activation motifs (ITAM) prevents the contractile engulfment by the macrophages and gives a “don't eat me” signal to the innate immune system, a new signal that cancers seem to use to evade detection and destruction by the immune system [16].

##### TSPs

are extracellular matrix calcium-binding glycoproteins regulating cell motility, proliferation, and differentiation [17]. Five isoforms of TSPs are currently known; TSP-1 is the first endogenous ligand identified for CD47. It is secreted by vascular and inflammatory cells that regulate cellular functions. It is a major component of platelet α granules and is released upon activation [18]. Binding of TSP-1 through its C-terminal binding domain (CBD) peptide 4N1K, to extracellular IgV of CD47, is responsible for several biological processes such as inflammation, immune responses, cellular proliferation, apoptosis, adhesion, and migration [19]. This binding also has a role in thrombus formation through platelet activation and aggregation as well as homeostasis. The antiangiogenic properties of TSP-1 come from blocking the nitric oxide (NO) pro-survival responses in endothelial and vascular smooth muscle cells (VSMC) [20]. TSP1-CD47 has been reported to inhibit NO signaling in several cell types and consequently stimulate osteoclastogenesis [21]***.*** Disruption of CD47-TSP-1 interaction by TSP-1-blocking antibodies or down-regulation of CD47 on tumor cells by RNA interference abrogates tumor-induced osteoclast formation in multiple myeloma [22]. It has also been shown that disruption of the TSP-1/CD47 interaction has positive outcomes in cancer therapy [23].

##### Integrins:

CD47 was initially found to interact with α_v_β_3_ integrins [1]. However, it was then shown to also interact with other integrins such as α2β1—which has a role in migration and proliferation of smooth muscle cells [24]*,* α4β1—which has a binding domain to *N*-terminal domain of TSP-1 and TSP-2 and a role in adhesion of reticulocytes [25], α5β1—which is involved in chondrocytes mechano-transduction [26], and α6β1—which has a role in fibrillar β-amyloid-mediated microglia activation and phagocytosis [27].

##### VEGFR-2:

Considering its ubiquitous expression, CD47 plays a central role in the tumor microenvironment (TME) [4]. Binding of CD47-TSP-1 inhibits VEGFR-2 phosphorylation and its downstream signaling, without affecting VEGF binding. Inhibition of tumor angiogenesis could be the rationale of the potential therapeutic effect of CD47 as an anti-cancer therapy [4].

##### CD36

 is necessary for inhibition of some angiogenic responses of TSP-1 and is therefore assumed to be the receptor that mediates its anti-angiogenic activities after CD47 binding [8, 20].

##### Fas (CD95)

is expressed by various cell types and mediates apoptosis as part of the normal physiology and in response to various stimuli. The modulation of the level of expression of CD47 influences Fas-mediated apoptosis [28]. For example, Jurkat T cells lacking CD47 are resistant to apoptosis mediated by Fas but are killed by Fas-ligand upon expression of CD47 [10]. The association of Fas with the extracellular IgV domain of CD47 causes downstream activation of Fas-mediated apoptosis pathway. Subsequently, cytochrome c release from the mitochondria with loss of mitochondrial membrane potential and DNA cleavage. Thus, CD47 enhances downstream activation of caspase-dependent death pathways [10].

#### Interaction with intracellular ligands

##### G_i_ proteins

Many of the cellular effects of CD47 are mediated by Gi proteins. It is hypothesized that binding of CD47 (which holds five transmembrane spanning segments) to integrins that have two transmembrane domains forms a seven-transmembrane receptor complex. Ligation of this complex with an adhesive ligand or TSP could in turn activate G-protein signal transduction. Other evidence suggests that CD47 ligation, even without interaction with integrin, could activate G-protein signaling [29].

##### BNIP3

(BCL-2 homology 3-only protein 19 kDa interacting protein-3) is a pro-apoptotic member of the BCL-2 family [6]. It mediates CD47-induced T-cell apoptosis triggered by TSP-1, which is independent of caspase activation and cytochrome c release and characterized by a plasma membrane and mitochondrial damage occurring prior to chromatin condensation and DNA fragmentation [6, 18].

## Role of CD47 in hematological neoplasms

The following section will address the role of CD47 in hematological malignancies. The ubiquitous high level of CD47 gene expression in many hematological malignancies and its impact on the clinical outcomes and prognosis will be discussed. Diverse therapeutics, including monoclonal antibodies to CD47 or SIRPα, receptor decoys, and bispecific antibodies that block the CD47-SIRPα axis have demonstrated significant anti-tumor activity in preclinical models of various hematological neoplasms [30, 31]. This supported many preclinical and clinical trials that have investigated the safety and the efficacy of anti-CD47 in treating most of the hematological malignancies.

### Role of CD47 in NHL

Many studies have demonstrated the important role of CD47 in various types of non-Hodgkin lymphomas.

#### B-cell lymphomas:

CD47 is variably expressed in many subsets of B-cell NHL including diffuse large B-cell lymphoma (DLBCL), chronic lymphocytic leukemia (CLL), follicular lymphoma (FL), mantle cell lymphoma (MCL), and marginal zone lymphoma (MZL) [32]. In cases of DLBCL, CD47 expression is significantly higher in activated B-cell like (ABC) compared with germinal center B-cell like (GCB) subtypes and does not carry independent prognostic value within GCB and ABC subtypes. However, CD47 expression remains an independent predictor of disease progression in multivariate analysis with the international prognostic index for DLBCL. In cases of CLL, higher levels are associated with unmutated Ig heavy chain variable regions, an adverse prognostic factor. In MCL, CD47 level is significantly correlated with the proliferative index [32].

Interestingly, the dissemination of tumor cells in a Burkitt lymphoma (BL) model was dependent on the level of CD47 expression. Also, CD47 expression was increased in disseminated tumoral cells in control DLBCL xenograft animals, as well as in peripheral blood lymphoma cells compared with lymph node tumoral cells from NHL patients (although note peripheral blood and lymph node cells were not harvested from the same patients), further supporting a role for CD47 in the dissemination of B-NHL tumor cells [33].

DLBCL and FL cancer cells express a high level of CD47 with an antiphagocytic signal that allows them to evade the immune response [34]. A recent phase 1b trial showed that the monoclonal antibody Hu5F9-G4 in combination with rituximab induced a high rate of tolerable and durable complete responses in heavily pretreated patients with rituximab-refractory DLBCL and FL. Blocking CD47-SIRPα interaction induced the phagocytic cells to recognize and attack cancer cells and this response was augmented by adding rituximab (please see “Targeting of CD47-SIRPα using antibodies” section for more details) [34].

#### T-cell lymphomas (TCL):

 Jain et al. demonstrated in their study that CD47 is widely but variably expressed in human TCL cells lines and primary samples. In addition, major histocompatibility complex class 1 (MHC-1) was widespread among the lymphoma cell lines and suppressed the phagocytosis of tumor cells. Monoclonal antibodies targeting CD47-SIRPα interaction (SRF231, B6H12, MOPC-21) promoted phagocytosis of TCL cells and improved outcomes in patient-derived xenograft and immunocompetent TCL mouse models. There was a synergistic effect when combining with an antibody targeting MHC class I (W6/32) [35]. Flow cytometry performed on 25 patients with Sezary syndrome (SS) showed higher CD47 expression in tumor cells compared with benign T-cells from the same individual. Patients with high expression of CD47 had 6.5 times shorter overall survival (OS) than patients with low expression (median OS, 84.0 vs 12.9 months; *P* < .001) [36].

#### Primary effusion lymphoma (PEL):

 Goto et al. showed that CD47 is highly expressed by flow cytometry in PEL cell lines. Furthermore, therapeutic knockdown of CD47 in PEL cell lines and anti-CD47 antibodies promoted the phagocytosis of the lymphoma cells in vitro*.* Treatment of a xenograft mouse model with anti-CD47 antibody (B6H12.2) inhibited ascites formation and organ invasion [37].

#### Role of CD47 in leukemia

##### Lymphoblastic lymphoma/Acute lymphoblastic leukemia (LBL/ALL:

 CD47 was increased in B-ALL and T-ALL patient samples and the expression level correlated with worse outcomes. Higher CD47 expression also correlated with worse OS in a cohort of patients and in an independent gene expression dataset. In vitro, ex vivo, and in vivo experiments demonstrated the therapeutic effect of anti-CD47 antibody B6H12.2 and BRIC126 against human ALL cells [30].

CD47 expression by immunohistochemistry (IHC) and by quantitative reverse transcriptase-polymerase chain reaction (qRT-PCR) was significantly higher in lymph nodes involved by T-LBL/ALL compared with reactive lymph nodes. The levels were significantly higher in patients ≤25 years old. The overall 1-year survival rate was lower in patients with high levels of CD47 or programmed cell death ligand (PD-L1) proteins by IHC and mRNA by qRT-PCR. High expression of CD47 and PD-L1 proteins were independent prognostic factors by multivariate analysis. There was a positive correlation between CD47 and PD-L1 mRNA expression but not protein expression [38]. Another study in transgenic mouse models and MYC-induced T-ALL cell lines showed that MYC regulates CD47 and PD-L1 mRNA and its protein expression by binding to its promoter genes. Therefore, CD47 and PD-L1 upregulation could have a direct role in MYC-driven tumorigenesis, which has implications for other MYC-driven malignancies [39].

##### Acute myeloid leukemia (AML):

 CD47 is highly expressed in AML leukemic stem cells and AML cells. Interestingly, CD47 mRNA had lower expression in cases harboring t (8;21), a favorable risk translocation, whereas higher expression is strongly correlated with FLT3-ITD mutations that confer worst survival in AML with normal cytogenetics. High CD47 expression was an independent prognostic factor for poor OS in two adult cohorts of AML patients. Monoclonal antibodies against CD47 (B6H12.2 and BRIC126) enabled phagocytosis of AML leukemic stem cells in vitro and inhibited their growth in mice models [40, 41].

Magrolimab, anti-CD47 antibody, has been shown to be effective and tolerated when combined with azacitidine in AML and myelodysblastic syndrome (MDS) patients [42].

Pietsch and colleges generated a panel of anti-CD47 antibodies using hybridoma and phage display technologies. They evaluated their activity and affinity to SIRPα of human and cynomolgus monkey CD47, as well as their capacity to induce hemagglutination and platelet aggregation. The majority of these mAbs were potent SIRPα blockers, only few did not induce hemagglutination or platelet aggregation. They found that 10 mg/kg of IgG1 C47B157, C47B161, and C47B222 suppressed leukemia growth in their xenograft human AML mice models, C47B222 showed the most consistent activity. However, a non-human study showed significant anemia after two doses of 1 mg/kg, raising concerns about potential toxicity [43].

Brierley et al. reported a decline in Hb levels in all patients (median Hb change, −1.0 g/dL; range, 0.4–1.6) and increased transfusion requirements with the administration of anti-CD47 Hu5F9-G4 in a phase 1 study of nineteen patients with relapsed/refractory AML (NCT02678338). Eighteen developed a newly positive direct antiglobulin test (DAT); however, there was no laboratory evidence of hemolysis [44]. The NCT02641002 trial, a phase 1 trial using the monoclonal anti-CD47 CC-90002 in treating relapsed and/or primary refractory AML and high-risk MDS patients was terminated because it did not offer a sufficiently encouraging profile for further dose escalation/expansion.

These early results suggest that perhaps different classes of anti-CD47 therapeutic drugs, or combinations with other types of anti-leukemia therapies, should be explored in future AML studies.

#### Role of CD47 in multiple myeloma

An analysis of patient samples with multiple myeloma (MM) (171 patients) and monoclonal gammopathy of undetermined significance (MGUS) (18 patients) showed that CD47 mRNA levels increased with progression from MGUS to MM [45]. Interestingly, comparison of samples in 6 patients with extramedullary lesions showed that extramedullary plasma cells show little to no expression of CD47 compared to bone marrow plasma cells, suggesting that the role of CD47 is less important once cells migrate outside the bone marrow environment. This particular study did not find an association between CD47 expression and OS [46]. However, a very recent study has reported that CD47 MM cells had remarkably higher CD47 expression than other cell populations in the bone marrow. These findings indicate that CD47 is specifically expressed on MM and can be used as a potential therapeutic target. They also showed that blocking of CD47 using an anti-CD47 antibody-induced immediate activation of macrophages and eliminated MM cells in the 3D-tissue engineered bone marrow model, as early as 4 h [47].

Kim et al. [48] demonstrated in their study of 37 patient samples that myeloma cells express higher levels of CD47 by flow cytometry compared with patient-matched normal bone marrow cells. CD47 was also consistently expressed in various myeloma cell lines. Blocking CD47 with B6H12 antibodies increased phagocytosis of myeloma cells in vitro. In their mice engrafted MM models, anti-CD47 Ab B6H12 inhibited the growth of myeloma cells and led to significant tumor regression and eradication, with a remission rate of 72% vs 19% in the control group at 6 weeks. Irradiation of mice before myeloma cell transplantation inhibited the efficacy of anti-CD47 antibodies delivered 2 weeks after radiation, and analysis of the bone marrow, spleen, and liver revealed that progeny of radiation-sensitive hematopoietic cells, and not only radiation-resistant resident macrophages, were necessary for CD47 antibody-mediated therapeutic effect [48].

Rastgoo and colleagues reported that high CD47 expression by IHC was associated with 17p (p53) deletions (*p* = 0.0407) and elevated beta-2 microglobulin level (*p* = 0.0323) in a cohort of 74 newly diagnosed MM patients [49]. High H-scores were associated with shorter median progression-free survival (PFS) and OS. This was also confirmed by analysis of CoMMpass database of 676 patients where high CD47 mRNA levels correlated with decreased PFS and OS. CD47 expression was also higher in drug-resistant cell lines, suggesting a potential role in drug response in MM [49].

## Therapeutic approaches for targeting CD47 in hematological malignancies

As described in the previous sections, CD47 is overexpressed in a variety of hematological cancers and plays a significant role in tumor evasion of immune surveillance [50]. There are many efforts in trying to target CD47 at different points in its signaling pathway for the purpose of cancer treatment. We will focus on pre-clinical and clinical studies targeting the CD47 axis in hematological malignancies. Tables 1 and 2 summarize the data.

**Table 1:** Therapeutics targeting CD47 in hematological malignancies

Drug	Type/target	Target	Phase	Malignancy type	References
B6H12.2	Humanized anti-CD47-IgG4	CD47-SIRP α	Cell lines, animal model	B-cell NHL, PEL, MM	[30] [37, 48]
*Hu5F9-G4*	Humanized anti-CD47-IgG4	CD47-SIRPα	Phase 1	B-cell NHL AML	[34, 44], NCT02678338
*CC-90002*	Humanized anti-CD47-IgG4	CD47-SIRPα	Phase 1	B-cell NHL	[51], NCT02367196
C47B157 C47B161 C47B222	Humanized anti-CD47-IgG1	CD47-SIRPα	Animal model	AML	[43, 44]
SRF231	High-affinity anti-CD47 Antibody	CD47-SIRPα	Cell lines, animal model, phase1	B-cell NHL	[52], NCT03512340
*ALX148*	High-affinity SIRPα-Fc Antibody	CD47-SIRPα	Cell lines, animal models	B-cell NHL	[53]
*W6/32*	Antibody	CD47-MHC-1	Cell lines, animal models	TCL	[35]
4N1K and 4N1	CD47-SIRPα Peptide agonist	CD47-SIRPα	Cell lines	B-cell NHL	[11, 54]
TTI-621	Human SIRP*α*-Fc fusion IgG1 Peptide Agonist	CD47-SIRPα	Phase 1	MDS, AML, MM, B-ALL, T-ALL, SS	[36, 55] NCT02663518
TTI-622	Human SIRP*α*-Fc fusion IgG4 Peptide Agonist	CD47-SIRPα	Phase 1	NHL, MM, c-HL	[56], NCT03530683
PKHB1	CD47 Peptide Agonist	CD47-SIRPα	Cell lines, Animal model	T-ALL	[57, 58]
SEN177	Antibody	QPCTL on CD47	Cell lines	BL	[59]
MiR-708	Mi-RNA	CD47-SIRPα	Cell lines, animal model	T-ALL	[60]
MiR-155	Mi-RNA	CD47-SIRPα	Animal model	MM	[49]
NI1701+ Rituximab	BsAb	CD47, CD19, CD20	Cell lines, animal model	NHL	[61]
Anti-CD47 and PD-L1	BsAb	CD47-PD-L1	Cell lines, animal model	T-LBL/ALL	[62–65]
Anti-(CD47&CD20)	BsAb	CD47-CD20	Cell lines, animal model	NHL	[66]
LicMABs	BsAb	CD47-CD33	Cell lines, animal model	AML	[67]

**Table 2:** Currently recruiting clinical trials targeting CD47 in hematological malignancies

NCT	Phase	Malignancy type	Agent used	Target
NCT02953509	1&2	NHL	Hu5F9-G4+Rituximab	CD47 + CD20
NCT03527147	1	NHL	Hu5F9-G4+Rituximab	CD47 + CD20
NCT03248479	1	AML-MDS	Hu5F9-G4+Azacitidine	CD47 + DNA methyltransferase
NCT02367196	1	H. Neoplasm	CC90002+Rituximab	CD47 + CD20
NCT02663518	1	H. Neoplasm	TTI-621+Rituximab	CD47 + CD20
NCT02890368	1	Mycosis Fungoides	TTI-621+Anti PD-L1	CD47 + PD-L1
NCT03530683	1	MM	TTI-622+Carfilzomib	CD47+ 26S proteasome
NCT03013218	1	NHL	ALX148+ Rituximab	CD47 + CD20
NCT03512340	1	H. Neoplasm	SRF231	CD47
NCT03763149	1	Lymphoma	IBI188	CD47
NCT03717103	1	NHL	IBI188 + Rituximab	CD47+ CD20

### Targeting CD47-SIRPα

As described earlier, CD47 and SIRPα are both transmembrane proteins that interact with each other as well as with the TME. SIRPα is abundant in myeloid cells such as macrophages and dendritic cells (DCs). Murata et al. reviewed the CD47- SIRPα axis in depth and its potential applications for cancer therapy [50].

**Fig. 2:**
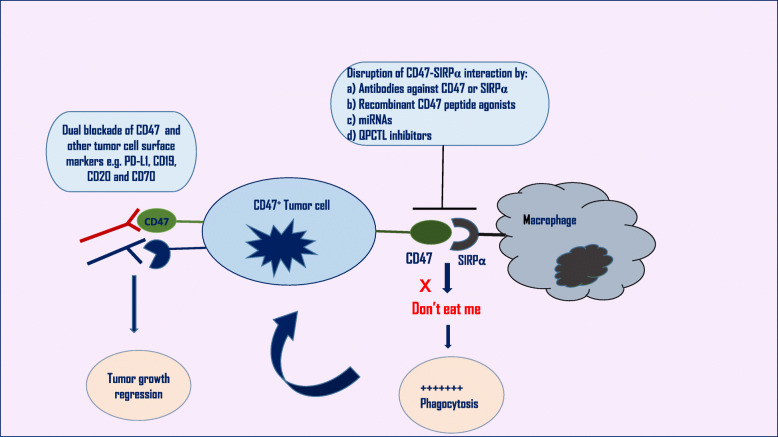
Therapeutic approaches for targeting CD47 in hematological malignancies. Blockade of CD47-SIRPα interaction (blocking “don't eat me” signal) through different approaches including antibodies, recombinant peptide agonists, miRNAs, and QPCTL inhibitors enhances the activity of phagocytes toward tumor cells (activating “eat me” signal). Dual blockade of CD47 and other hematological surface markers by antibody therapies is another approach to synergistically target tumor cells

A growing evidence that the blockade of CD47-SIRPα interaction “don’t eat me” signal enhances the activity of phagocytes toward tumor cells in vitro as well as in a variety of xenograft models of cancer [55]. This blockade promotes the stimulation of tumor-specific cytotoxic T-cells, macrophages, or DCs. Biological agents targeting CD47-SIRPα, such as antibodies (Abs), recombinant peptide agonists, and miRNAs have been tested in preclinical and clinical trials. Such targeting modulates both innate and acquired immune responses and is considered a promising strategy for cancer treatment [68]. Combinations of molecules targeting CD47 and other hematological surface markers were also extensively studied and have shown synergistic effects [56]***.*** Figure 2 summarizes different mechanisms of targeting CD47-SIRPα interaction.

#### Targeting of CD47-SIRPα using antibodies

##### Hu5F9*-*G4

(Hereafter, 5F9), a macrophage immune checkpoint inhibitor, promotes tumor cell phagocytosis by the innate immune response through blocking of the CD47-SIRPα axis [34]. The most common adverse event (AE) of this humanized antibody is anemia due to its non-selective inhibition of CD47 on aging red cells. A phase1b study of 22 patients with heavily pre-treated rituximab-resistant DLBCL and FL were enrolled in their study. A total of 50% of the patients had an objective response, with 36% having a complete response. Most AEs during treatment were of grade 1 and 2, and the most common treatment-related AEs were chills in 9 patients, headaches in 9 patients, anemia in 9 patients, and infusion-related reactions in 8 patients. The treatment-related hemolytic anemia could be managed by giving a small priming dose of Hu5F9-G4 to eliminate the aging red cells and select for younger red cells (lacking prophagocytic signals), followed by a higher maintenance dose [34]. These results corroborate the beneficial effects of adding anti-CD47 antibodies to lymphoma treatments, especially when most of AEs were tolerated.

##### CC-90002

is another humanized IgG4 anti-CD47 antibody that inhibits CD47-SIRPα interaction and enabled phagocytosis in cancer cell lines, including hematological cancer cell lines, as well as in solid tumor xenografts [69]. In a current phase 1 multicenter study (NCT02367196), CC-90002 was combined with rituximab to treat CD20-positive relapsed/refractory NHL. Out of 28 enrolled subjects, 24 were treated with escalating doses of CC-90002 and rituximab. Anemia was common, but there was no evidence of hemolysis. The most frequent grade 3/4 AEs were neutropenia (38%) and thrombocytopenia (21%). Twenty subjects discontinued the study, mostly due to progressive disease or death. Seven deaths occurred; 6 from progressive diseases (PD) and 1 due to an AE. The overall response rate was 13%, with a median duration of response of 3.9 months [51].

##### SRF231

is a fully humanized monoclonal anti-CD47 antibody produced by phage technology. Data presented in an abstract showed it selectively blocks CD47- SIRP α, promoting phagocytosis of cancer cells and sparing T-cells and RBCs in vitro. Coadministration of anti-CD20 antibodies and SRF231 enhanced tumor phagocytosis in MM and lymphoma animal models [52]. A phase 1 study to evaluate the safety and tolerability of SRF231 as a monotherapy in solid and hematological malignancies is ongoing, but there are no clinical results available yet (NCT03512340).

##### B6H12.2

, a CD47 antibody, promoted phagocytosis of tumor cells in ALL cell lines. When ALL cells were coated with B6H12.2 ex-vivo, tumor cell engraftment in vivo was nearly abolished. Furthermore, it eliminated the mice engrafted ALL cells in peripheral blood, bone marrow, liver, and spleen. It could also induce long-term remissions in the treated mice [30]. Additionally, B6H12.2 was found to inhibit the extranodal dissemination of human BL Raji cells in mice models and a DLBCL xenograft model [33].

##### ALX148

antibody has been generated by fusing an inactivated human IgG1 Fc with a modified SIRPα D1 domain. It blocks the CD47-SIRPα interaction and stimulates both innate and acquired immune responses, promoting DCs, macrophage, and T-cell responses. Combination of ALX148 with obinutuzumab (anti-CD20 Ab) in a mouse subcutaneous xenograft model of MCL resulted in enhanced tumor growth inhibition compared with either drug alone (*p* < 0.01), whereas the combination of ALX148 with rituximab-enhanced tumor growth inhibition and survival compared with rituximab alone in Raji B-cell lymphoma tumors (*p* < 0.001 and *p* < 0.0001). ALX148 did not cause hemagglutination of human red blood cells and had a good safety profile in a non-human primate toxicity study [53].

##### KWAR23

, another blocking Ab to human SIRP α, was inert when administered on its own, but enhanced the effect of rituximab in a human BL xenograft model [31].

#### Targeting of CD47-SIRPα using CD47 peptide agonists

##### 4N1K and 4 N1

 are mimicry peptides of CD47. 4N1K is analogous to the C-terminal part of TSP-1. 4 N1 and 4N1K are non-selective and may bind to receptors independent from CD47 [70]. 4N1K was shown to induce apoptosis in monocytes; surviving cells could differentiate to DCs, but those had decreased capacity to secrete inflammatory cytokines IL-12 and TNFα [54]. The 4N1K peptide triggered caspase-independent apoptosis in Jurkat T cells through a G protein-mediated reduction of c-AMP levels and inhibition of protein kinase-A (PKA) [11].

##### SIRPα-IgG1 Fc

(TTI-621**)** is a checkpoint inhibitor that binds to human CD47 in tumor cells. As a decoy receptor protein, it is composed of *N*-terminal V domain of human SIRPα linked to the Fc region of human immunoglobulin G1 (IgG1). It prevents inhibitory signals to macrophages by binding to Fc gamma receptors (FcgR) and enhancing phagocytosis of tumor cells. It effectively controlled the growth of aggressive cancer cells and promoted macrophage phagocytosis of 77% (23/30) of hematological tumor cell lines. There was also enhanced phagocytosis in 97% (32/33) of primary samples from patients with hematological malignancies (AML, MDS, MM, B-ALL, and T-ALL). This effect appears to be tumor cell-specific. The antitumor activity of SIRP***αF***c (TTI-621) was detected in mouse AML and B-cell NHL xenografts models. There was an only minimal binding of human erythrocytes, which is of interest given the concerns for hemolytic anemia with CD47 mAbs [55].

The open-label phase 1a clinical trial (NCT02663518) used SIRPαFc (TTI-621) in five patients with SS. The patients were pretreated and had high leukemic counts. After single-dose infusion of TTI-621, 4 of 5 patients had a decrease in their count and a rapid decrease of lactate dehydrogenase (LDH). Additional experiments showed that SIRPαFc increased the phagocytosis of ex vivo Sezary cells extracted from patient blood, but not non-malignant lymphocytes [36].

##### SIRPα-IgG4 Fc

(TTI-622) is a soluble recombinant fusion protein created by directly linking the *N*-terminal CD47 binding domain of human SIRPα with the Fc domain of human immunoglobulin (IgG4). TTI-622 acts by binding human CD47 and preventing it from delivering an inhibitory “don’t eat me” signal to macrophages [56]. A clinical trial testing TTI-622 (NCT03530683) is currently recruiting patients. It will be conducted in two phases for patients with refractory lymphoma and MM. Phase 1a is a dose-escalation phase and phase 1b is a combination treatment phase. In phase 1b, TTI-622 will be given to subjects with CD20-positive NHL, classic Hodgkin lymphoma (cHL), and MM, in combination with other anti-cancer drugs to define their safety and efficacy. The combination treatments are TTI-622 + rituximab in DLBCL and indolent NHL patients, TTI-622 + PD-1 inhibitor nivolumab in cHL, and TTI-622 + Proteasome-inhibitor carfilzomib + dexamethasone in MM. The results are not available yet. Lin and colleges [71] demonstrated the efficacy of TTI-622 in their animal models of DLBCL, BL, and MM. TTI-622 monotherapy showed improved OS and partial tumor growth regression. Combination therapy of TTI-622 with daratumumab (anti-CD38 antibody) and cetuximab (anti-EGFR antibody) potentiates its therapeutic efficacy. TTI-622 associated risk of anemia is minimal because it does not induce hemagglutination and has minimal binding to human erythrocytes [71].

##### PKHB1

is a TSP1-derived CD47 agonist peptide. PKHB1 induced caspase-independent and calcium-dependent cell death in T-ALL cell lines. It was also tested in an immunocompetent leukemic mouse model, where it induced immunogenic cell death by promoting DC maturation and the release of several damage-associated molecular patterns (DAMPs) such as calreticulin (CRT), heat shock proteins 70, 90 (HSP70 and HSP90), ATP, and high-mobility group box-1 (HMGB1). This was further confirmed by giving mice a prophylactic vaccine of tumor cells previously treated with PKHB1, which prevented tumor engraftment. In tumor-bearing mice, PKHB1 treatment could induce complete tumor regression in most mice [57]. Treatment of tumor-bearing mice with the tumor cell lysate obtained from PKHB1-treated cells could similarly induce tumor regression, improve OS, and protect the mice from further relapse [58].

#### Targeting of CD47-SIRPα using microRNA-based approaches

Studies showed that microRNAs (miRNAs) encapsulated into liposome-protamine-hyaluronic acid nanoparticles could regulate CD47 expression levels, and results from animal models of solid tumors suggested that they could represent potential cancer strategies [72, 73]. In hematological malignancies, Huang et al. used Target Scan prediction and studied the microarray expression patterns of miRNA in cases of human ALL. They selected five miRNAs (miR-15a/b, miR-128, miR-143, and miR-708) with different expression patterns at diagnosis and relapse or remission of T-ALL. Among those five, miR-708 showed the most significant effects on luciferase activity of CD47, suggesting potent inhibition. This finding was also confirmed by miR-708 overexpression and knockdown experiments in various cell lines. Analysis of primary patient T-ALL samples confirmed the inverse correlation between miR-708 levels and CD47 mRNA expression. A combination of miR-708 and CD47 antibodies caused greater phagocytosis and apoptosis than either agent alone, suggesting a synergistic effect [60].

Our group recently used bioinformatics strategies to identify miR-155 as a potential regulator of CD47 [49]. We used luciferase assay, overexpression, and functional rescue assays to further demonstrate that miR-155 targets CD47 and that increased miR-155 expression could consequently induce phagocytosis of drug-resistant MM cells. Moreover, we showed that miR-155 also targets the tumor necrosis factor-alpha-induced protein 8 (TNFAIP8), which is a negative regulator of apoptosis. Downregulation of TNFAIP8 sensitized the MM cells to the protease inhibitor bortezomib (BTZ) and promoted the apoptosis of myeloma cells. Restoration of miR-155 levels in their xenograft mice model of resistant MM combined with Bortezomib suppressed tumorigenesis and extended overall survival. IHC showed an increase in the apoptotic index compared to Bortezomib or miR-155 mimics alone. These results support the fact that downregulated miR-155 plays an essential role in the pathogenesis of drug resistance in MM through upregulation of both CD47 and TNFAIP8, and that it represents a promising therapeutic strategy [49].

#### Targeting QPCTL

Glutaminyl peptide cyclotransferase-like protein (QPCTL) is a newly discovered major protein component of the CD47 signaling pathway. It is essential for forming pyroglutamate on the CD47-SIRPα binding site. Logtenberg and colleges showed in their study that SEN177, a glutaminyl cyclase inhibitor, enhanced antibody-dependent cellular phagocytosis of anti-CD20-treated BL (Raji) cells to the same or greater extent than combining rituximab with 12C4 (SIRPα-blocking agent) of B6H12. In a syngeneic peritoneal tumor model of HER2-expressing Ba/F3 pro-B cells, there was a significant increase of neutrophil-mediated selective killing of QPCTL- (and CD47-) deficient cells treated with anti-HER2. This anti-tumor activity was like a full genetic deficiency of CD47. QPCTL inhibitors represent a promising anti-cancer strategy that could potentially avoid the antigen sink of other anti-CD47 drugs due to CD47 expression on erythrocytes and other cells [59].

### Dual blockade of CD47 and other hematological malignancies markers

As presented in previous sections, dual blockade of CD47 and other hematological surface markers already targeted by antibody therapies was shown to have synergistic effects. This section will address bispecific antibodies that target two markers at the same time. It has been hypothesized that these antibodies could be more specific for neoplastic cells and avoid side effects such as hemolytic anemia or thrombocytopenia that result from the relatively high expression of CD47 on red blood cells and platelets [61].

#### Dual blockade of CD47 and PD-L1

As previously described, CD47 regulates the innate immunity and overexpression on tumor cells and TME promotes evasion of the immune surveillence [5]. On the other hand, PD-L1, is normally expressed on antigen-presenting cells (APCs) and pathologically expressed on diverse types of cancer cells and TME [74]. It interacts with the programmed cell death-1 (PD-1) receptor expressed on the surface of cytotoxic T-cells, thereby inhibiting T-cell-mediated immunity. Boussiotis extensively reviewed the PD-1/PD-L1 axis and its potential role in cancer therapy [74]. Previous studies showed enhanced anti-tumor effect with dual targeting of both CD47 and PD-L1 in melanoma and colon carcinoma [75, 76]. Lian and colleagues designed an Ep-CAM (epithelial cell adhesion molecule) liposome containing both CD47 and PD-L1 arms. It targets high-Ep-CAM cancer cells and effectively knocks down both CD47 and PD-L1 proteins. This therapeutic effect was proved in solid tumor cell lines and mice lung cancer models [77]. To our knowledge, this strategy has yet to be tested in hematological malignancies but given the known role of PD-L1 in numerous hematological malignancies, it likely represents a viable line of investigation [62–65].

#### Dual blockade of CD47 and CD20

A study in B-NHL xenograft mouse models of BL cell line, as well as primary human DLBCL and FL, showed a synergistic therapeutic effect when combining anti-CD47 antibodies (B6H12.2 and BRIC126) with rituximab and allowed long-term disease-free survival [32].

Piccione and colleagues developed a bispecific antibody (BsAb) that selectively binds to CD20 and CD47 by using the dual-variable-domain immunoglobulin (DVD-Ig) format. Two variants of CD20-CD47 DVD-Ig were formed either by short (SL) or long (LL) linker sequence between the variable domains. In human cell lines, the BsAb showed selective binding to tumor cells with dual expression of CD20 and CD47. The SL form binds poorly to erythrocytes compared to anti-CD47 (B6H12.2) alone, which is advantageous to avoid the potential issue of hemolytic anemia. In mice engrafted human NHL, the SL form showed potent antitumor activity and prolonged survival compared with anti-CD47 or rituximab alone, recapitulating the synergistic effect seen with a combination of anti-CD47 and rituximab [78]. They obtained similar results by grafting the SIRPα *N*-terminal Ig domain onto rituximab, who played the role of a tumor-specific scaffold [66].

#### Dual blockade of CD47 and CD19

Buatois et al. [61] designed a BsAb, NI-1701, that specifically and effectively targeted both CD19 and CD47. Flow cytometry confirmed strong binding of B-cells, without significant binding of T-cells or erythrocytes, and only limited binding of platelets. This antibody showed anti-tumor activity in vitro for cell lines of B-cell NHL (DLBCL, BL), ALL, and (CLL) and in vivo cell-derived xenograft BL model and patient-derived xenograft ALL model. It showed a 4.2 and 52-fold increase in the phagocytic activity against Raji cells when compared to anti-CD19 and anti-CD47 monovalent antibodies respectively. NI-1701 also showed a more potent anti-tumor effect than either monovalent antibodies alone in mice. NI-1701 was superior to rituximab monotherapy, but co-administration of NI-1701 and rituximab showed a synergistic effect, leading to significant tumor growth regression in mice models and increased survival compared with single agents and controls. NI-1701 also demonstrated a potent therapeutic effect in a variety of B-cell NHL and B-ALL primary patient samples and a patient-derived mouse model of B-ALL. It showed a favorable safety profile in cynomolgus monkeys [61].

#### Dual blockade of CD47 and CD70

CD70 shows only limited expression in normal cells but is expressed on most B-cell malignancies and in multiple myeloma, as well as other solid malignancies. It could represent a target in CD20-negative malignancies. Therefore, Ring and colleagues developed a bispecific anti-human CD70/KWAR23 antibody. It showed synergistic effect in renal carcinoma cell lines compared to anti-CD70 vorsetuzumab and anti-CD47 drugs alone or in combination, however its therapeutic effect did not outperform combination of the individual drugs when tested in a Burkitt lymphoma mouse model [31]. This suggests additional studies would be needed to better understand the function of this bispecific antibody.

## Insights and challenges in targeting CD47 in hematological malignancies

CD47 is a target of interest and researchers are still trying to figure out what is the best way to target this pathway. Several anti-CD47 targeting agents have been developed and tested in many preclinical and clinical trials over a long-time scale. Such targeting successfully modulates both innate and acquired immune responses against tumor cells [79]. Despite promising results from most of these studies on the impact of anti-CD47 agents, challenges were found to be in selectivity, efficacy, and safety profile. Recent studies are trying to alleviate these challenges proving that CD47 targeting is a novel anti-cancer approach [80].

### Specificity of anti-CD47 targeting agents

To minimize or avoid cross-reactivity and damage of normal cells due non-selectivity of anti-CD47 agents while exerting anti-cancer effects is a challenge that requires consideration while designing future anti-CD47 therapies [81]. Ho et al. produced high-affinity CD47-ectodomain antagonist to increase the antibody-dependent phagocytosis [82]. Moreover, Sim and colleges [83] discovered high-affinity pan-mammalian and pan-allelic antibodies against SIRPα. Given that older erythrocytes are more susceptible to phagocytosis, future studies of CD47-SIRPα targeting should consider patient age [84].

Fusion proteins such as SIRPα-IG1 Fc (TTI-621) was developed to avoid damage to normal cells by fusion of the *N*-terminal V domain of human SIRPα to the human IgG1 Fc region. At low therapeutic levels, it showed minimal binding affinity to human erythrocytes while exerting enough tumor binding [55].

Furthermore, combination therapies were developed to achieve increased tumor specificity and decreased toxicity to CD47-expressing non-malignant cells. For example, anti-CD47 antibodies (BRIC126 or B6H12) combined with anti-CD20 rituximab resulted in NHL ablation in xenograft models [32, 78]. Enhancement of the anti-tumor effects of high-affinity SIRPα-CD47 antibodies obtained when combined with tumor-specific antibodies [85]. Anti-SIRPα antagonists have also been combined with tumor-opsonizing antibodies such as rituximab showed anti-tumor efficacy in vitro [82] and in xenograft lymphoma and colon cancer models [86]. Similarly, targeting CD47 and PD-L1 [75, 76].

The development of BsAb improved the specificity and reduced the cytotoxicity of anti-CD47 agents. NI-1701, a BsAb that targets CD47 and CD19 was designed for B-cell lymphoma and refractory leukemia [61]. Other bispecific agents (LicMABs) have been produced by the binding domain of SIRPα to a tumor-targeting antibody such as anti-CD33 promoted elimination of AML tumor cells [67].

Novel methods for specific targeting CD47 and its ligands on tumor cells have been proved in recent studies. These included drug delivery vehicles such as CD47-conjugated nanoparticles [87] or quorum-sensing bacteria [88]. Such delivery resulted in T cell and macrophage induced phagocytosis of tumor cells by blocking CD47 and reduced tumor progression [87, 88]. Other nanoparticles as magnetic iron oxide have been developed as vehicles for selective simultaneous delivery of anti-CD47 antibodies and gemcitabine for the treatment of pancreatic cancer without cytotoxicity [89]. Mitomycin A-loaded nanoparticles showed downregulation of CD47 expression in xenografted mice [90]. Davis et al. combined anti-CD47 antibodies with nanoparticles to target ovarian cancer cells [91]. No update information about using such vehicles in targeting CD47 in hematological malignancies.

### Efficacy of anti-CD47 targeting agents

The potency and the therapeutic effects of anti-CD47 agents varied between different studies according to the type of cancer, type of antibodies, stage of tumor, type of tumor model, drug pharmacokinetics, acquired drug resistanc,e and the state of the immune system [80]. Drug resistance and unsatisfactory results were in most cases related to tumor heterogeneity, tumor microenvironment changes, drug inactivation, decreased drug absorption, and epigenetic changes [92].

### Toxicity and safety profile of anti-CD47 targeting agents

Given that CD47 is ubiquitously expressed by normal of the hematopoietic system [93] such as RBCs [94] and platelets [95], potential adverse events using anti-CD47 antibodies as cancer therapeutics include anemia and thrombocytopenia. Buatois et al. [61] showed that Hu47F9-G4 alone or in combination with other antibodies may cause accidental killing of normal hematopoietic cells. To alleviate this adverse effect, one study [34] proposed to give short priming low-dose of Hu5F9-G4 in combination with rituximab to selectively eliminate the aged red blood cells, followed by long-term treatment. The toxicity of anti-CD47 antibodies is proved to be Fc-dependent; SIRPα-Fc fusion proteins give this toxicity while high-affinity SIRPα generations don’t [85, 96, 97]. These high-affinity variants bind to CD47 with a greater potency compared with wild type SIRPα [85]. They showed regression of solid tumors and hematologic malignancies in preclinical trials but not yet in clinical trials [85, 98].

## Conclusion and future perspectives

At the end of this review, we can conclude that CD47 is a novel promising target for cancer therapy. It is overexpressed in various hematological malignancies and plays a role in tumor dissemination. Strategies targeting the CD47-SIRPα axis, such as antibodies, inhibitory peptides, and miRNAs, demonstrated promising results for the treatment of hematological neoplasms. Combinations of CD47 inhibitory strategies and other anti-cancer therapies such as anti-CD20, anti-CD19, and others showed promising results suggesting a synergistic therapeutic effect. Early clinical phase 1/2 studies have shown encouraging therapeutic effects with tolerable AEs. Various strategies, such as priming with small doses to eliminate aging RBC, as well as many newly developed BsAbs, are being investigated trying to circumvent the hemolytic anemia and thrombocytopenia that can occur as a result of the expression of CD47 on red blood cells and platelets. Additional clinical trials are required to determine the clinical efficacy of these strategies. Moreover, certain aspects of targeting CD47 have not been fully investigated in hematological malignancies, such as the interaction between CD47 and TSP1, dual blockade of CD47 and PD-L1, targeting the QPCTL as proposed by Logtenberg and colleagues as well as the recently described nanoparticles delivery methods. This opens potential new avenues to target CD47 in hematological malignancies. Future advances in cancer screening would define which type and stage of cancer that could be treated with a specific type or types of anti-CD47 targeting agents.

## Data Availability

Data sharing is not applicable to this article as no datasets were generated or analyzed during the current study.

## Abbreviations

ABC: Activated B-cells
Abs: Antibodies
AE: Adverse Events
ALL: Acute lymphoblastic leukemia
AML: Acute myeloid leukemia
ATP: Adenosine triphosphate
BIT: Brain immunoglobulin-like molecule with tyrosine-based activation motifs
BL: Burkitt Lymphoma
BM: Bone marrow
BNIP3: BCL2 Nineteen kD interacting protein 3
BsAbs: Bispecific antibodies
c-AMP: Cyclic adenosine monophosphate
CBD: C-terminal binding domain
CD47: Cluster designation or cluster of differentiation 47
CLL: Chronic lymphocytic leukemia
CRT: Calreticulin
DAMPs: Damage-associated molecular patterns
DAT: Direct Antiglobulin Test
DC: Dendritic cells
DLBCL: Diffuse large B-cell lymphoma
DVD-Ig: Dual-variable-domain immunoglobulin
Ep-CAM: Epithelial cell adhesion molecule
FcgR: Fc gamma receptors
FL: Follicular lymphoma
GCB: Germinal center B-cells
GM-CSF: Granulocyte-macrophage colony-stimulating factor
HMGB1: High-mobility group box 1
HSCs: Hematopoietic stem cells
HSP: Heat shock protein
IAP: Integrin-associated protein
ICD: Immunogenic cell death
IHC: Immunohistochemical staining
IL-12: Interleukin 12
IL-6: Interleukin 6
ITAM: Immunoreceptor tyrosine activation motifs
ITIM: Immunoreceptor tyrosine inhibitory motifs
KD: Kilo-Dalton
LBL: Lymphoblastic Lymphoma
LDH: Lactate dehydrogenase
LL: Long linker sequence
MCL: Mantel cell lymphoma
MDS: Myelodysblastic Syndrome
MEK: Mitogen-activated protein kinase
MGUS: Monoclonal Gammopathy of Undetermind Significance
Mi-RNA: Micro-RNA
MM: Multiple myeloma
MZL: Marginal zone lymphoma
NCT: National Clinical Trial
NHL: Non-Hodgkin’s lymphoma
NO: Nitric oxide
NTD: Non-tolerated dose
OS: Overall survival
PB: Peripheral blood
PD-1: Programmed cell death-1
PD-L1: Programmed cell death ligand-1
PEL: Primary effusion lymphoma
PFS: Progression-free survival
PKA: Protein kinase A
PTPases: Protein tyrosine phosphatases
QPCTL: Glutaminyl peptide cyclotransferase-like
Q-RT-PCR: Quantitative reverse transcriptase-polymerase chain reaction
SH2: Src Homology region 2
SHP-1: Src homology phosphatase-1
SHP-2: Src homology phosphatase-2
Si-RNAs: Silencing RNAs or small interfering RNAs
SIRP: Signal regulatory protein
SL: Short linker sequence
SS: Sezary syndrome
T-LBL: T-lymphoblastic leukemia
TNFAIP8: Tumor necrosis factor alpha-induced protein 8
TNFα: Tumor necrosis factor α
TSP: Thrombospondins
VEGF: Vascular endothelial growth factor
VEGFR-2: Vascular endothelial growth factor receptor-2
VSMC: Vascular smooth muscle cells
